# Challenges in corneal endothelial cell culture

**DOI:** 10.2217/rme-2020-0202

**Published:** 2021-08-12

**Authors:** Rintra Wongvisavavit, Mohit Parekh, Sajjad Ahmad, Julie T Daniels

**Affiliations:** 1 Institute of Ophthalmology, University College London, London, UK; 2 Faculty of Medicine & Public Health, Chulabhorn Royal Academy, Bangkok, Thailand; 3 Moorfields Eye Hospital NHS Foundation Trust, London, UK

**Keywords:** cell culture, corneal endothelial cell markers, corneal endothelial cells, corneal endothelial cell therapy

## Abstract

Corneal endothelial cells (CECs) facilitate the function of maintaining the transparency of the cornea. Damage or dysfunction of CECs can lead to blindness, and the primary treatment is corneal transplantation. However, the shortage of cornea donors is a significant problem worldwide. Thus, cultured CEC therapy has been proposed and found to be a promising approach to overcome the lack of tissue supply. Unfortunately, CECs in humans rarely proliferate *in vivo* and, therefore, can be extremely challenging to culture *in vitro*. Several promising cell isolation and culture techniques have been proposed. Multiple factors affecting the success of cell expansion including donor characteristics, preservation and isolation methods, plating density, media preparation, transdifferentiation and biomarkers have been evaluated. However, there is no consensus on standard technique for CEC culture. This review aimed to determine the challenges and investigate potential options that would facilitate the standardization of CEC culture for research and therapeutic application.

The cornea is a transparent, avascular tissue on the outermost surface of the eye. The sensory function of the eye depends on the transparency of the cornea, which determines the quality of vision and, ultimately, quality of life. Transparency depends on both the outer and inner integrity of the corneal epithelium and corneal endothelium (CE), respectively. As the corneal endothelial cells (CECs) do not have regenerative potential *in vivo*, they must be maintained throughout life. Damage or dysfunction of these cells could lead to partial or total blindness. Various pathologies cause CEC dysfunction, including viral infection, intraocular surgery and Fuchs' endothelial dystrophy (FED). FED, the most common etiology of corneal endothelial dysfunction, is a primary indication for corneal transplantation [1–3]. This disorder affects up to 4% of those over 40 years old in the USA [4], and up to 35% of all corneal transplants performed in the United Kingdom are due to FED [5].

Cornea transplantation is a standard treatment for patients with corneal endothelial diseases. In the past, FED was treated with penetrating keratoplasty (PK), which is a transplantation procedure that replaces the diseased cornea with a full-thickness donor corneal graft. The donor tissue is held in place with sutures. Although PK has many benefits, it has several risks and complications. Surgical-related complications, such as suture-induced astigmatism and glaucoma, can lead to poor vision. Graft-related complications involve corneal graft rejection, which remains a possibility for life. Furthermore, there is a shortage of cornea donors worldwide [6]. Patients are often denied a sight-saving corneal graft due to the lack of tissue donors. This shortfall has led to considerable interest in refined transplant methods such as endothelial and lamellar keratoplasty. The number of patients who benefit from these methods is increasing because one corneal graft can be split into two and used for more than one transplant – anterior lamellar keratoplasty utilizes the anterior stroma and posterior lamellar keratoplasty or endothelial keratoplasty (EK) facilitates endothelial cell transplantation.

EK is a relatively new procedure for treating endothelial diseases. Being a sutureless technique, patients are not at risk of suture-induced astigmatism, and their functional vision may recover within days to weeks. Furthermore, the graft rejection rate is lower than PK [7]. EK consists of Descemet stripping automated endothelial keratoplasty (DSAEK) and Descemet's membrane endothelial keratoplasty (DMEK). The difference between the two techniques is graft composition. DSAEK grafts contain a part of the stroma with Descemet's membrane (DM) and endothelium with an overall thickness of 100–200 μm [8], whereas DMEK grafts only have DM and endothelium with an overall thickness of 14–20 μm [9].

Although corneal transplantation is the standard treatment for patients with corneal endothelial diseases, the shortage of cornea donors remains a significant problem. The number of patients requiring treatment is much greater than that of donors [6], and their quality of life deteriorates as they wait for treatment. Hence, several techniques have been proposed and examined, such as tissue substitutes using tissue-engineering methods and CEC culture *in vitro*. However, the attempt to promote CEC proliferation both *in vitro* and *in vivo* is challenging. The CECs are presumed to lack the ability to proliferate because of cell cycle arrest at the G1 phase. Understanding CEC biology and using suitable promoting factors can thus provide a way to overcome this issue. Several techniques for culturing endothelial cells *in vitro* [10,11] have been discussed; however, only one successful clinical trial has been performed, thus limiting the understanding of potential therapeutic possibilities [12]. Additionally, several factors including molecular, cellular and epidemiological play an important role in determining a successful therapeutic application. Thus, this review highlighted the challenges faced with CECs in terms of cellular profile, proliferative capacity and downstream analysis.

## Development of CECs

CE develops when neural crest cells migrate and localize between the corneal epithelium and the lens at 6 weeks of human gestation. The cells spread and loosely form a monolayer at the posterior corneal surface [13]. At 8 weeks of gestation, the cells are tightly formed and present endoplasmic reticulum. However, lateral cell membranes do not develop tortuous line of tight junctions, which means the cells do not mature at this stage [14]. CEC differentiation and maturation are present after birth. The cells develop tight junctions and close intracellular spaces [15]. CE is characterized as one layer of cells with DM underneath them, and this membrane has two layers, namely, anterior banded layer and posterior nonbanded layer. Anterior banded DM is recognized as multiple-banded filaments with a thickness of 2–4 μm in fetal corneas at 12 weeks of gestation [16]. The thickness is constantly maintained throughout life. After birth, the CECs secrete the extracellular matrix (ECM) and form the posterior nonbanded layer. Deposition of nonbanded filaments significantly continues with age [17]. ECM components found in both anterior banded and posterior nonbanded DMs in an adult cornea are collagen types IV and VIII, fibronectin and laminin [17,18].

Any interference of neural crest migration and differentiation causes congenital anterior segment malformations. Peters' anomaly is representative of anterior segment dysgenesis during eye development. Multiple gene loci have played a role in mesenchymal cell differentiation including *PITX2*, *FOC1*, *PAX6* and *CYP1B1*. These genes are established as a cause of Peters' anomaly [19]. Clinical findings vary because of the large spectrum of anterior segment dysgenesis. However, defects in the posterior stroma, DM and endothelium are found in classic Peters' anomaly and cause congenital corneal opacity.

The study by Bahn *et al.* evaluated the relationship between CEC density and corneal diameter in mammalian corneas at the early postnatal stage. The infant and adult corneas from cats, cows, dogs, rabbits and humans were examined. The results showed that CEC density was about 2500 cells/mm^2^ in every species after birth. At the early postnatal period, corneal diameter and surface area rapidly increased in all of the species, except humans. The corneal surface area increased by 543% in dogs but only 15% in humans. In terms of CEC density, the decline in cell density is found in all species. However, the total number of cells per cornea increased in nonhuman species but decreased in humans with a loss of 45%. The reason was from the mitotic activity found in mammalian CECs during corneal maturation [20]. The results from this study showed that the number of human CECs declined after birth without cell mitosis due to the cells being arrested in the G1 phase of the cell cycle [21,22]. The number of cells also declined with age and then became stable at the age of 50 years. The decline rate is approximately 0.6% per year [23,24]. CECs cannot proliferate *in vivo*. The reason for the lack of progression in the cell cycle is still unknown, but it may be caused by the contact inhibition of the cells and the lack of growth factor stimulation [23,25].

## The CECs & endothelial cell function

**Figure 1.**
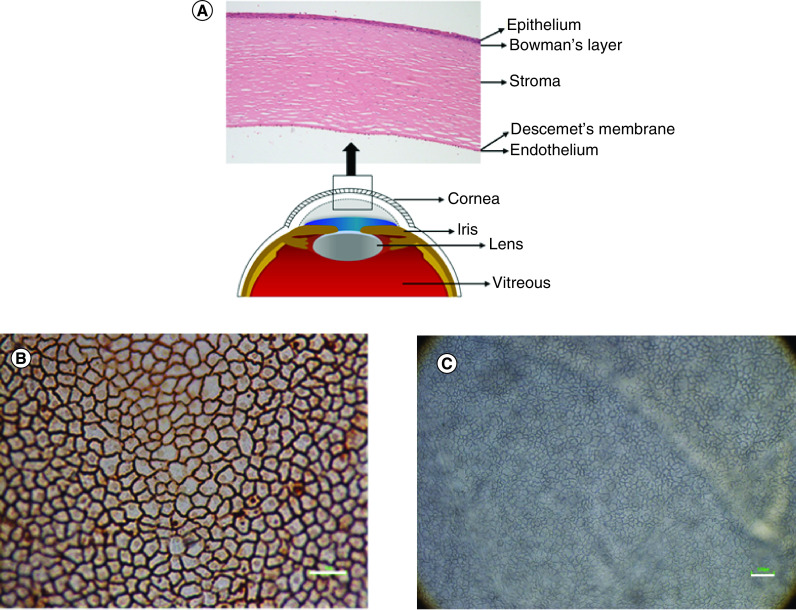
Structural anatomy of the human cornea and corneal endothelial cells. **(A)** The cornea consists of five layers; epithelium, Bowman's layer, stroma, Descemet's membrane and endothelium. **(B)** Corneal endothelial cells presented hexagonal morphology after staining with alizarin red. **(C)** Corneal endothelial cells observed under light microscope. Scale bar = 100 μm.

CE is the innermost layer of the cornea with a thickness of 5 μm [26]. It consists of CECs that form a monolayer of hexagonal cells on the posterior corneal surface. CECs can be observed after alizarin red staining and using inverted light microscope on the whole human corneal tissue (Figure 1). ZO-1 is an essential submembranous protein of the tight junction lining along the apical side of the cells. The presence of ZO-1 at the cell membrane shows hexagonal shapes of CECs [27]. The encoded protein from ZO-1 may be involved in signal transduction at cell–cell junction.

CECs play several roles in maintaining corneal homeostasis, transparency and thickness, and they regulate aqueous humor flow into and out of the stroma. Their function is to not only serve as a barrier, which is facilitated by the cells' tight junction complex, but also act as an active ion and solute transporter. CECs allow leakage of solutes and nutrients from the aqueous humor to provide nutrition for stromal keratocytes and corneal epithelial cells. At the same time, CECs pump water via active transport from the stroma and into the aqueous to maintain systematic homeostasis, which in turn results in appropriate corneal hydration and transparency. The active transport of fluid out of the stroma depends on the sodium/potassium (Na^+^/K^+^)-ATPase pump [28], which is a plasma membrane protein pump that mediates the ATP-dependent transport of Na^+^ and K^+^ across the membrane, leading to low internal Na^+^ and high internal K^+^ concentrations. Na^+^ is cotransported with sugar, water and amino acids, which are essential nutrients of the cells. The pump density is 1.5 × 10^6^ pump sites per cell. These pump sites are expressed at the basolateral membrane of the CECs [29]. This mechanism regulates corneal homeostasis. Loss of this function could result in corneal edema, leading to partial or complete blindness.

## CEC markers

**Figure 2.**
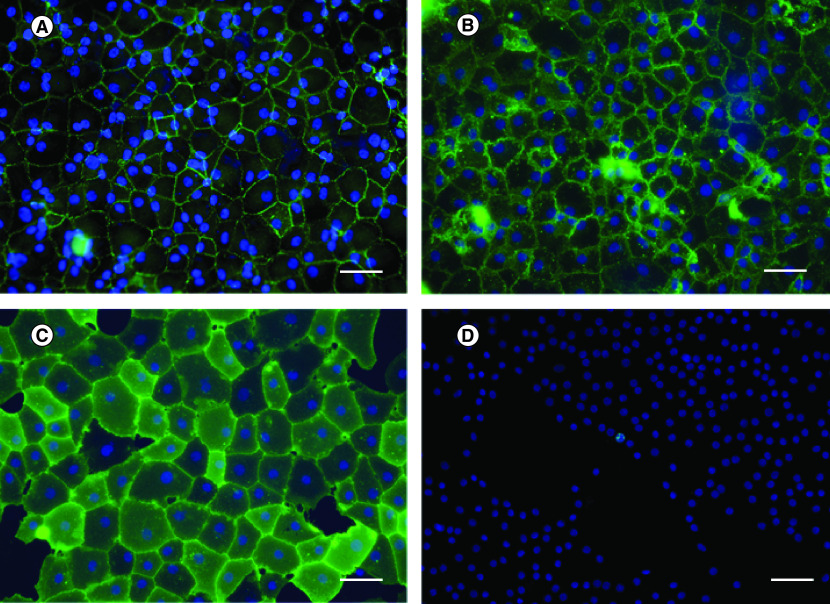
Immunocytochemistry using different biomarkers. Human corneal endothelial cells were stained in green for **(A)** ZO-1, **(B)** 1A3, **(C)** 2A12 and **(D)** Ki-67. Nuclei were counterstained in blue. Hexagonal shape of corneal endothelial cells was clearly observed after stained with cell surface marker antibodies. Cell proliferation marker, Ki-67, was used to evaluate number of cells which have proliferative capacity. Scale bar = 100 μm.

Cell characterization is used to identify specific characteristics of the cells, including cell morphology, presence of certain protein and cell function. Several markers used in CEC characterization are listed in Table 1. The intercellular tight junction protein is located at the apical surface of the cells. ZO-1 antibody was applied in immunocytochemistry (ICC) and immunohistochemistry (IHC). The results were presented as a zig-zag line at the apical cell membrane; thus, the hexagonal shape of the cells was observed (Figure 2) [30,31]. CDH2, which is found at the lateral cell membrane, also presented hexagonal shapes of CECs [27]. Cell morphology obtained from ZO-1 and CDH2 markers was clearly observed via confocal microscopy. When comparing the specificity of ZO-1 and CDH2 in CEC characterization, CDH2 showed more specificity than ZO-1. CDH2 was the only marker exclusively found in CECs, whereas ZO-1 was also found in corneal epithelial cells [27]. Nevertheless, CD166 and Prdx-6 markers, present at the lateral membrane, were used to identify cell morphology (Figure 2). IHC result presented a flower-shaped appearance of interdigitating foot processes in contact with DM [27]. CD166 played a role in cell–cell adhesion, which led to the formation of cell aggregation, in various cell types. However, the function of CD166 in CECs remained unknown. Ding *et al.* proposed the new monoclonal antibody (mAb) TAG-1A3 and TAG-2A12 [32]. The study compared the commercial anti-CD166 antibody against mAb TAG-1A3 and found that both antibodies were comparable in function based on IHC, ICC and flow cytometry (FCM). Meanwhile, TAG-2A12 presented a better result than mAb Prdx-6. It was found that mAb Prdx-6 was bound to intracellular Prdx-6 and cross-reacted with corneal epithelial cells and stromal fibroblasts. Simultaneously, TAG-2A12 was specifically bound to cell surface Prdx-6 without cross-reaction. A recent study by Parekh *et al.* demonstrated the use of TAG-1A3 and TAG-2A12 in ICC [31]. The cells presented hexagonal and polygonal shapes under confocal microscopy. In conclusion, the phenotype of CECs could be obtained from IHC and ICC using markers including ZO-1, CDH2, TAG-1A3 and TAG-2A12.

**Table 1.** Corneal endothelial cell markers.

CEC markers	Location	Function	Method	Remark	Ref.
Na^+^/K^+^-ATPase	Basolateral membrane	Active transport pump	WB, ICC, IHC, RT-PCR	Found in epithelial cells, TM	[27,30,31,33,34]
ZO-1	Apical submembraneous protein of the tight junction complex	Intercellular tight junction	WB, ICC, IHC, RT-PCR	Found in epithelial cells	[27,30,31,33]
VDAC3	Mitochondrial membrane channels	Mitochondrial permeability transition pore	RT-PCR	None	[33]
SLC4A4	Plasma membrane	Na-HCO_3_ co-transporter, membrane transport protein	RT-PCR	None	[33,34]
CLCN3	Plasma membrane	Membrane transport protein	RT-PCR	None	[33]
COL4A2	Extracellular matrix	Produced by CECs	RT-PCR	None	[34]
COL8A1, COL8A2	Extracellular matrix	Produced by CECs	RT-PCR, QPCR, WB	DM component	[34–36]
CDH2	Transmembrane	Adherents junctions	IHC, RT-PCR	Specific for CECs	[27,30,34]
CD98	Plasma membrane	Membrane transport protein	FCM	None	[30]
CD166	Basolateral transmembrane protein	NA	ICC, RT-PCR, FCM, IHC, IP	Found in epithelial cells, stromal cells, TM	[27,30–32,27]
CD340	Plasma membrane	NA	FCM	None	[30]
Integrin α3β1	Basal membrane	NA	IHC	Found in epithelial cells	[27]
CD56 (NCAM)	Subapical lateral membrane	Cell–cell adhesion during embryonic development	IHC, FCM	Found in stromal cells, TM	[27,37]
Prdx-6	Lateral plasma membrane	Role of antioxidant	ICC, FCM, IP, IHC	Found in TM	[27,31,32,38]
CD248	Transmembrane	NA	FCM	None	[37]
SLC4A11	Transmembrane	Na^+^/OH^−^ co-transport, Na^+^-independent H^+^ (OH^-^) transport, NH_3_ transport	QPCR	None	[35,39]
CYYR1	Transmembrane	NA	QPCR	None	[35]

CEC: Corneal endothelial cell; DM: Descemet's membrane; FCM: Flow cytometry; ICC: Immunocytochemistry; IHC: Immunohistochemistry; IP: Immunoprecipitation; NA: Not available for corneal endothelial cells; QPCR: Quantitative PCR; RT-PCR: Reverse transcription PCR; TM: Trabecular meshwork; WB: Western blot.

To identify the CEC function, Na^+^/K^+^-ATPase marker is widely used. Previous studies have used the expression of Na^+^/K^+^-ATPase and ZO-1 as typical markers of monolayer CECs [31,33]. However, the cells strongly expressed both the antibodies, and they became confluent and form a tight junction. The expression decreased when the cells were removed from a monolayer [37].

The screening of cell surface markers on human CECs using FCM showed CD98, CD166 and CD340 on the cell membrane [30]. Meanwhile, the result from the PCR showed transport proteins – SLC4A4 [33,34], CLCN3 [33], VDAC3 [33], SLC4A11 [35] and CYYR1 [35]. Ki-67, a non-CEC-specific cell proliferation marker, has also been widely used in human CEC culture to evaluate the percentage of proliferative cells *in vitro*.

### Markers of ECM produced by CECs

COL4A2, COL8A1 and COL8A2 were also found in the ECM produced by CECs [34]. The results from quantitative PCR showed that COL8A2 was highly expressed in CECs from both young and old donors. The expression was presented in CECs both *in vivo* and *in vitro* with no detection from stromal fibroblasts [35]. This specificity could be applied for CEC characterization.

## Challenges of culturing CECs *in vitro*

As described earlier, the cells do not proliferate *in vivo*, and the treatment of every patient is difficult, considering that the supply of human corneal donor tissues is significantly limited. It therefore becomes important to find ways to increase the number of human CECs *in vitro* for potential therapeutic purposes. Many studies have shown that growth factors or kinase inhibitors could induce the proliferative capacity of CECs *in vitro*. However, several challenges need to be addressed to achieve a successful cell culture.

## Donor factors

The primary source of CECs is the cornea donors. Therefore, donor characteristics such as age, gender, post-mortem time, cause of death, cell density and tissue preservation can significantly influence the success of cell culture.

### Age of cornea donors

The primary requirement for a successful CEC culture is a source of viable, proliferative cells. Human donor corneas with high endothelial cell density (ECD), which is a common characteristic of tissues from young donors, are usually used for transplantation. Hence, the tissues from older aged donors with low ECD are usually left for research. The differences between young and older aged donor corneas are listed in Table 2.

**Table 2.** Evaluation of different parameters observed when the cells are cultured from young and older aged donor tissue.

Parameter	Young donor	Old donor	Ref.
Proliferative capacity	47%	23%	[40]
Cell senescence	Late	Early	
Endothelial cell density	3000 cell/mm^2^	2700 cell/mm^2^	[41]
Availability	Low	High	
Passage capacity	Maintain cell morphology at passage 4	Loss cell morphology at passage 4	[42]

Corneal donor information from SightLife Eye Bank (2012–2016) presented a correlation between donor age and ECD [41]. A total of 39,679 donor corneas were analyzed, and a simple linear regression analysis between ECD and donor age was performed. The result showed that there was a strong correlation between ECD and age (p < 0.001). The ECDs at aged 20–40 and 50–70 years were about 3000 and 2700 cell/mm^2^, respectively. Meanwhile, multiple regression showed no relationship between sex and ECD.

The study from Joyce and Senoo [40] categorized the cornea based on donor age as follows: young (<30 years old) and old (>50 years old). An *ex vivo* wound-healing study monitored the relative ability of human CECs, from young and older donors, to enter and complete the cell cycle that was analyzed using Ki-67. The expression of Ki-67 protein indicated that cells were active in the late G1 phase through to the M phase completion of the cell cycle, which represented cell proliferation. Human CECs from young donors expressed Ki-67 36 h after wounding, whereas the CECs from older age donors delayed their proliferation until 48 h. Nevertheless, the percentage of proliferative cells in the young donor group (47%) was greater than in the older donor group (23%) [40].

Peh *et al.* revealed that human CECs from young donors could be expanded up to the third passage while maintaining their polygonal cell shape [11]. Meanwhile, the cells that expressed CEC characteristic markers, ZO-1 and Na^+^/K^+^-ATPase, were detected by ICC. At the fourth and fifth passages, the cells lost their unique polygonal morphology to take up an elongated morphology. Another study demonstrated the heterogeneity of human CECs, which is obtained from young and older donors. Human CECs were collected from corneoscleral rims, redundant after transplantation. The cells were cultured until the fourth passage, and the cell area was analyzed. The results showed that cultured human CECs from old donors presented greater heterogeneity [42].

As the CECs from the older donor groups undergo early senescence during the culture period, Parekh *et al.* demonstrated that forced attachment of these cells could increase the proliferation and migration of human CECs *in vitro* [43]. The study described the use of hyaluronic acid (HA), a viscous gel typically used in cataract surgeries, to reduce damage to CECs *in vivo* and ROCK inhibitor as forced attachment factors. The CECs, cultured in combination with HA and ROCK inhibitor, showed complete confluence within 10–15 days. This technique showed an improvement in cell adhesion efficiency due to forced attachment as observed using Vinculin (focal adhesion marker) positivity.

**Figure 3.**
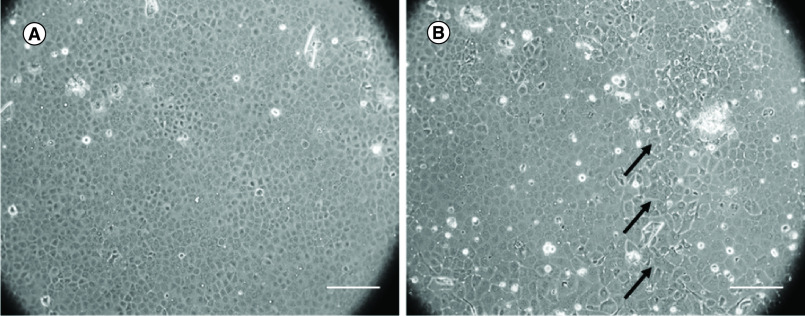
Human corneal endothelial cells from young and older donors. **(A)** Human corneal endothelial cells from the young donor showed homogeneous hexagonal morphology. **(B)** Human corneal endothelial cells from older donor showed polymegathism and pleomorphism. The polymorphic cell is marked with black arrows. Scale bar = 250 μm.

Human CECs obtained from young donors showed better proliferative capability (Figure 3A) and retained their homogeneity than those from older donors (Figure 3B). The use of human CECs from young donors was preferred to maximize these benefits for *in vitro* cell culture. However, the corneas from young donors were difficult to obtain for cell culture as most are destined for clinical corneal transplantation. Using the corneas obtained from older donors, the protocol for culturing these cells becomes more challenging for *in vitro* studies. For *in vitro* studies, culturing cells from old aged donors becomes far more challenging.

### Difference between central & peripheral endothelium

A study by Parekh *et al.* investigated the difference between culturing central (8.25 mm) and peripheral (2.75 mm) CECs from older aged donor tissues. The study found that the number of central and peripheral endothelial cells increased by 8.25 and 16.5%, respectively. However, the cell structure, morphology and Ki-67 positivity did not differ significantly when the cells were cultured from either area. Nevertheless, it was identified that although there are relatively few CECs available for cell culture from the peripheral region, they can still be used for cell culture due to their high proliferative capacity [44]. Following which, Parekh *et al.* [45] demonstrated successful culture of human CECs obtained from older donors. Here, cells were collected from 2.75 mm at the peripheral area of the clinical-grade cornea (PE) and 11 mm at the central area of the research-grade cornea (CE). For the clinical-grade tissues, the endothelium was taken 9.5 mm from the central for preloaded DMEK graft preparation. The remaining peripheral corneas were used in this study. The results showed that the number of cells harvested from the PE and CE before plating were 1457 and 2093 cell/mm^2^, respectively. After culture, average cell densities at confluence were 2510 and 2352 cell/mm^2^ in the PE and CE groups, respectively. The confluency period and cell morphology did not differ between groups. The cells similarly expressed ZO-1, CD166 and Prdx-6 as reflected by ICC [45]. It was assumed that the cells from these two conditions had a similar propensity for confluency period and cell characteristics. This study highlighted an alternative source of human CECs for cell culture *in vitro*. The cells from PE become an optional resource. Peripheral human CECs, with proliferative potential, have been referred to as human corneal endothelial progenitor cells (HCEPs) [46–48], which can be differentiated into mature human CECs. Moreover, HCEPs were found at the human transition zone, the posterior limbus of corneal periphery, where they expressed neural crest cell markers, p75 neurotrophin receptor, SOX9 and FOXC2 [46], as well as stem/progenitor markers (Sox2, Lgr5, CD34, Pitx2 and telomerase) [49]. Another study found specific stem cell markers (nestin, ALP and telomerase) at the trabecular meshwork (TM) and the transitional zone between TM and the peripheral area of the cornea using immunofluorescence. After wounding, peripheral CECs presented additional stem cell markers, Oct-3/4 in the TM and Wnt-1 in both TM and transitional zones. Furthermore, differentiation markers (Pax6 and Sox2) were also found at the peripheral area of the corneal endothelium [47]. The study by Whikehart *et al.* found positive telomerase activity at the peripheral and intermediate areas of the normal cornea. After wounding, they also found an increase in CEC proliferative marker expression (BrdU and TGF-β) [48].

### Preservation condition of tissues

Optisol-GS and organ culture are widely used to preserve donor cornea for transplantation. Optisol-GS is an intermediate-term hypothermic storage solution that preserves the corneal tissues at 4°C for 14 days. Meanwhile, organ culture preserves corneal tissues up to 28 days at 31°C. As organ culture contains serum, it allows constant metabolism of CECs during storage time compared with hypothermic storage that reduces the metabolic cell activity because of the temperature the tissues are preserved in [31]. The study from Kitazawa *et al.* evaluated the viability of donor CECs preserved in Optisol-GS [50]. The peripheral portion of the remaining corneas after transplantation was collected and assessed using propidium iodide, calcein-AM and Hoechst. The results showed that dead cell rates varied between donors from 0.6 to 10.5%, with a mean dead cell rate of 4.9% ± 3.3% [50]. Meanwhile, a recent study used trypan blue to identify the percentage of dead cells in donor corneas preserved in Optisol-GS and organ culture [31]. The percentages of dead cells were 9.33% ± 4.04% and 0.46% ± 0.35% in Optisol-GS and organ culture, respectively. The *in vitro* study also demonstrated successful culture of human CECs from different donor cornea preservation conditions (Optisol-GS and organ culture). The cells were cultured and examined for human CEC-associated markers, ZO-1, Na^+^/K^+^-ATPase, CD166, Prdx-6 and Ki-67. On day 9 after culture, the cells became confluent and expressed these markers [31]. Based on these results, it was prompted that the viability of CECs after preservation in organ culture is greater than in Optisol-GS. However, human CECs from different preservation conditions could yield successful cultures. The cells expressed human CEC-associated markers, which subsequently refer to the cell morphology, function and proliferation.

## Evaluation & isolation of human CECs

Before cell isolation, viability of CECs could be evaluated using trypan blue, which is a vital dye used to selectively stain dead cells. Parekh *et al.* applied 0.25% trypan blue on the endothelium surface and left for 20 s at room temperature. After washing with phosphate-buffered saline, the tissue was submerged in a hypotonic sucrose solution (~1.8%) to swell the intercellular borders. Using a reticule in an eyepiece of a microscope, the number of CECs was counted manually, and the cells with positive trypan blue staining were determined as dead cells [51]. Based on this study, trypan blue with hypotonic sucrose could be used to evaluate cell number and CEC viability before isolation. In addition, the viability and plating density of the isolated cells following enzymatic digestion can also be confirmed with trypan blue using a hemocytometer slide.

In terms of cell isolation, the peel-and-digest method by Peh *et al.* [11] has resulted in successful cultures. In brief, DM and endothelium are peeled from the cornea and digested by the enzymes. One difficulty observed while culturing human CECs was isolating them from DM because these cells exhibit strong adherence to ECM via the ECM collagens. However, several enzymes have been investigated such as trypsin [52], collagenase [11] and dispase or ethylenediaminetetraacetic acid (EDTA) [53,54].

To isolate human CECs from DM and ECM collagens using trypsin, a prolonged incubation period was needed that resulted in CEC degeneration rather than cell isolation [52]. Other studies used dispase or EDTA, followed by gentle pipetting [53,54]. This method was not only time consuming but also caused cell damage and cell number decrease.

Based on the fact that the main composition of ECM collagens, which help human CECs adhere to DM, is type IV collagen. Collagenase was applied for human CEC isolation. The compact of human CEC aggregates was presented after 2 h of incubation period, and intercellular junctions mediated by ZO-1 and cell-to-basement membrane interaction were maintained. After culture, the cells presented human CEC markers, Na^+^/K^+^-ATPase and ZO-1 [11].

**Figure 4.**
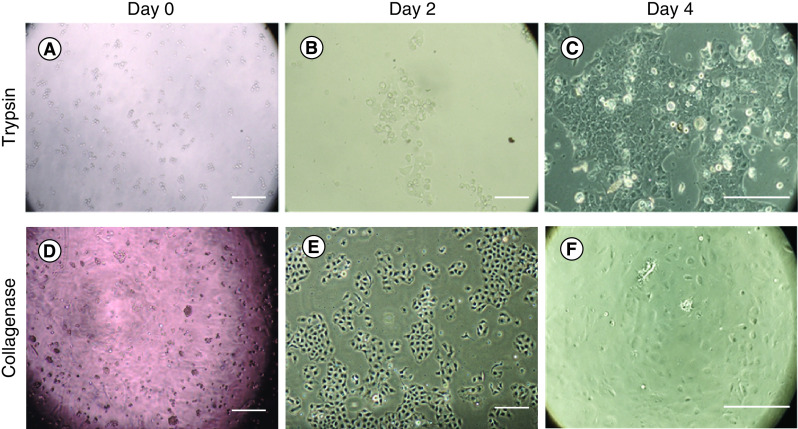
Human corneal endothelial cell isolation using trypsin and collagenase. At day 0, **(A)** the cells digested with trypsin presented smaller cell number compared with **(D)** collagenase. At day 2, **(E)** the cells digested with collagenase showed better proliferation than the cells digested with **(B)** trypsin. At day 4, **(F)** nearly 50–70% confluence was found in the collagenase group. Meanwhile, **(C)** around 30–40% cell confluence was found in trypsin group. Scale bar = 250 μm.

The most promising enzyme for isolating CECs was collagenase. Moreover, the use of collagenase helps isolate viable cells to maintain their intercellular junction and cell-to-basement membrane interaction. The number of viable cells after collagenase digestion was greater than after trypsin digestion followed by greater cell proliferation rate (Figure 4). The pros and cons are listed in Table 3.

**Table 3.** The difference between isolation of human corneal endothelial cells using trypsin and collagenase digestion techniques.

Parameter	Trypsin digestion	Collagenase digestion
Cell isolation	Enzyme digestion and isolation	Enzyme digestion and isolation
Mortality	Low	Low
Amount of cells obtained	High	High
Time required	Low	Moderate

## Cell plating density

A study by Peh *et al.* demonstrated a suitable cell plating density for maintaining CEC morphology [55]. The CECs obtained from young donors were cultured until passage 3. The cells from the third passage were seeded with different cell plating densities. CECs were seeded at 2500, 5000, 10,000 and 20,000 cells/cm^2^ for low, middle, high and high×2 plating densities, respectively. Cell morphology and proliferation were observed. For cell proliferation, CECs at low and middle plating densities showed greater proliferation rate than CECs at high and high×2 plating densities without statistical significance. However, CECs seeded at low and middle plating densities presented fibroblastic-like cell morphology with larger cell bodies than that at high and high×2 plating densities. CECs seeded at high and high×2 plating density presented homogeneous cell morphology with a compact hexagonal cell shape. Based on these results, it was noted that the optimal seeding density should be no less than 10,000 cells/cm^2^ for regular passage of CECs to maintain specific cell morphology [55]. This plating density was useful for a robust cell expansion strategy to obtain a sufficient number of CECs for tissue engineering or cell injection therapy.

In addition, other studies used different cell plating densities ranging from 15,000 to 25,000 cells/cm^2^. Lu *et al.* demonstrated the effect of stem cell conditioned medium (CM) on CEC proliferation using cell plating density of 25,000 cells/cm^2^ [33]. Meanwhile, a study on the effect of orbital adipose-derived stem cell (OASC)-CM on CEC proliferation used cell plating density of about 22,000 cell/cm^2^ [56]. The results from these studies showed that CECs could proliferate while maintaining their specific characteristics. Another study from Bartakova *et al.* seeded 15,000 cells/cm^2^ to monitor cell morphology and proliferation when cultured in different media [57]. The cells had proliferative capacity and maintained their typical morphology. From these studies, the diversity of cell plating density has been found. This range could result in different cell characteristics and future application reliability.

## Cell adhesion coating

In corneal tissues, human CECs adhere to DM via ECM components. DM consists of many ECM protein types, including collagen type IV, fibronectin and laminin [58,59]. Therefore, cell adhesion coating derived from the ECM proteins may be applied on the surface of tissue culture substrate before CEC plating to mimic a physiologically compatible environment for CECs.

Collagen IV is a basement membrane-specific collagen, which is found in the endothelial and epithelial layers [60]. The functions of collagen IV are essential for CECs such as maintaining the normal phenotype of CECs and preventing endothelial to mesenchymal transition (EndMT) [60]. An *in vitro* study of bovine CECs showed that the cells cultured on glass or polystyrene with uncoated surface or fibronectin and collagen I coating lost their phenotype. On the other hand, the cells cultured on glass or polystyrene with collagen IV coating presented polygonal morphology and positive ZO-1 at the cell borders [61]. Furthermore, collagen type IV may facilitate cell attachment and promote cell expansion *in vitro* [62].

A tissue-engineering approach using silk fibroin, precoated with collagen type IV, has also been evaluated for human CEC culture. Here, human CECs proliferated and grew to confluence with polygonal morphology. Meanwhile, human CECs cultured on noncoated silk fibroin could not reach confluence [63]. Thus, collagen type IV might play a primary role in maintaining the phenotype of human CECs. These studies examined not only collagen type IV but also other ECMs (e.g., fibronectin and laminin). Various types of cell adhesion coating (fibronectin, poly-D-lysine, collagen type I, fibronectin/collagen type I and FNC coating mix^®^ [Athena Enzyme Systems, MD, USA]) were applied and examined for their adhesive capability to CECs. The wells were precoated with different coatings followed by cell seeding; the cells were then rinsed with cell culture media. The number of cells was counted and compared between groups. Collagen type I, collagen type I/fibronectin and FNC coating mix significantly enhanced the spreading of human CECs to tissue culture plates. Moreover, the use of FNC coating mix reduced cell loss after rinsing. Around 100% of the cells remained on the culture plates. The percentages of the remaining human CECs in the collagen type I, fibronectin and collagen type I/fibronectin groups were about 90, 70 and 90%, respectively. Meanwhile, the percentage of human CECs in the noncoated culture plates was 67% [64]. From these results, the FNC coating mix was the most suitable cell adhesion coating for human CEC cultures. It enhanced human CEC adhesion and supported cell spreading on tissue culture plates. Many studies used the FNC coating mix, and their results showed effective human CEC proliferation [30,65]. The study on cell surface markers of human CECs after being cultured *in vitro* applied the FNC coating mix before cell plating. After the culture, the cells were collected for cell characterization. Human CECs showed positive staining for CD98, CD166 and CD340 using FCM [30].

From this evidence, the FNC coating mix and collagen type IV could be suitable cell adhesion coatings for cell cultures since they facilitated cell adhesion and promoted cell proliferation. The cells increased in numbers and became confluent while maintaining their specific cell characteristics. Although the FNC coating mix was widely used in cell culture studies, it was unsuitable for clinical studies because it contains animal-derived components. The chances of prion contamination or any potential disease of animal origin are of significant concern.

**Figure 5.**
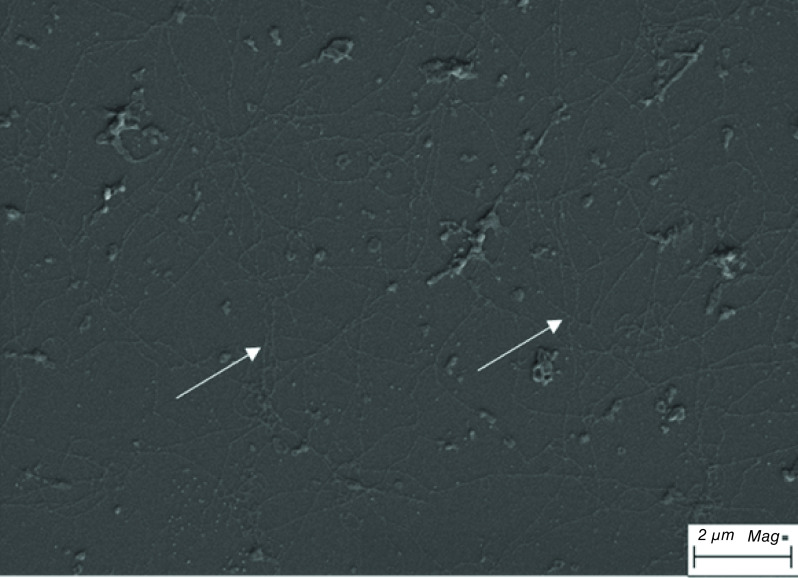
Extracellular matrix derived from HCECL-12 cell line. The extracellular matrix showed long collagen-like fibrillary structures marked with white arrow. Scale bar = 2 μm.

In a recent study, the ECM derived from the human CEC line (HCECL-12) was used for human CEC culture [51]. HCECL-12 cells were cultured for 9 days, and then, the cells were ruptured using ammonium hydroxide, leaving the released ECM on the culture surface that was investigated using a scanning electron microscope (Figure 5). Human CECs were cultured on FNC coating mix or ECM and were expanded until the second passage. The results showed that the cells cultured on ECM presented a significantly better proliferation rate and expressed the human CEC marker ZO-1 (detected by ICC) [51].

## EndMT

EndMT refers to endothelial cells that lose their specific markers and acquire mesenchymal characteristics. The key events of EndMT include disassembly of cell–cell junctions, loss of apical–basal polarity, reorganisation of actin cytoskeleton, change in cell shape, increase of cell motility, increase production of ECM proteins and change in gene expression [66]. EndMT is triggered by TGF-β, FGF-2 and IL-1β [67]. An *in vivo* study found that the normal human aqueous humor contains 2.3–8.1 ng/ml of total TGF-β [68]. Although TGF-β is found in the aqueous humor, most were present in an inactive form [69,70] and needed to be activated to induce and affect the cells *in vivo*. A previous study demonstrated the expansion of human CECs *ex vivo* by disrupting contact inhibition with EDTA and TGF-β as a supplement. The results showed that the cells lost their normal phenotype to EndMT, which was supported by ICC. The cells lost their typical positive staining of CDH2, ZO-1 and Na^+^/K^+^-ATPase [71].

### EndMT markers

The markers used for EndMT detection are listed in Table 4. The study by Okumura *et al.* presented the cell surface proteins obtained from FCM [30]. The EndMT markers were CD9, CD49e, CD44 and CD73. In addition to the ECM produced by EndMT, COL1A1 was also identified. Another research group reported that CD90, CD109 and CD248 could be used to identify fibroblastic changes during *in vitro* cell culture [37]. As detailed above, the detection of EndMT markers was based on PCR and FCM methods. Only the CD73 marker could be detected via ICC [30]. Furthermore, the role of these proteins on EndMT has not been clearly elucidated. Further studies are required to clarify the functions of these cell surface proteins, which might lead to EndMT prevention.

**Table 4.** Endothelial to mesenchymal transition markers.

EndMT markers	Location	Function	Method	Remark	Ref.
CD73	Plasma membrane	NA	ICC, RT-PCR, FCM	Found in stromal cells	[30,37]
CD9	Transmembrane	NA	FCM	None	[30,37]
CD44	Plasma membrane	NA	FCM	None	[30]
CD49e	Plasma membrane	NA	FCM	None	[30]
COL1A1	Extracellular matrix	Produced by fibroblastic cells	RT-PCR	None	[30]
CD90	Plasma membrane	NA	FCM	Found in stromal fibroblasts	[37]
CD109	Plasma membrane	NA	FCM	Involved in the TGF-β pathway	[37]
CD248	Transmembrane	NA	FCM	None	[37]

EndMT: Endothelial to mesenchymal transition; FCM: Flow cytometry; ICC: Immunocytochemistry; NA: Not available for endothelial to mesenchymal transition; RT-PCR: Reverse transcription PCR.

## CEC culture media

Different compositions of CEC culture media have been evaluated. Maintenance media consist of one or two basal media without exogenous growth factors. Proliferation media have one or two basal media with growth factors (Table 5).

**Table 5.** Corneal endothelial cell culture media.

**Media type**	Study	Basal media	Supplement	Special supplement	Ref.
Proliferation media	Parekh *et al.*	Ham's F-12 and M199	5% FBS, 20 μg/ml AA, 1% ITS, 10 ng/ml FGF2, 1% penicillin-streptomycin	10 μM ROCK inhibitor (Y-27632)	[45]
	Peh *et al.* Wahlig *et al.*	Ham's F-12 and M199	5% FBS, 20 μg/ml AA, 1% ITS, 10 ng/ml FBF2, 1% antibiotic/antimycotic	10 μM ROCK inhibitor (Y-27632)	[11,55,72,73]
	Okumura *et al.* Kinoshita *et al.*	Opti-MEM-I	8% FBS, 5 ng/ml EGF, 20 μg/ml AA, 200 mg/l calcium chloride, 0.08% chondroitin sulfate, 50 μg/ml gentamicin	10 μM ROCK inhibitor (Y-27632)	[12,30]
	Bartakova *et al.*	Opti-MEM-I	8% FBS, 0.5 mM AA, 200 mg/l calcium chloride, 5 ng/ml EGF, 50 μg/ml gentamicin, 1% antibiotic/antimycotic	100 μg/ml bovine pituitary extract, 20 ng/ml NGF	[57]
Maintenance media	Bartakova *et al.*	Human endothelial-SFM	4% FBS, 50 μg/ml gentamicin, 1% antibiotic/antimycotic		[57]
	Peh *et al.* Wahlig *et al.*	Human endothelial-SFM	5% FBS, 1% antibiotic/antimycotic		[72,73]

AA: Ascorbic acid; FBS: Fetal bovine serum; SFM: Serum-free media.

A dual media approach that utilizes both maintenance and proliferation media was proposed to prevent EndMT [72]. After cell isolation, CECs were cultured in maintenance media overnight and subsequently with proliferation media to promote cell growth. On the other hand, Bartakova *et al.* used proliferation media for cell expansion [57]. After the cells reached confluence, maintenance media was replaced to stabilize the cells before trypsinization. Results from these studies showed that CEC characteristics and functions were presented by CECs cultured in the dual media. Meanwhile, heterogeneous cell morphology with fibroblastic appearance was found in CECs cultured in proliferation media alone. The study by Parekh *et al.* demonstrated CEC culture using a single proliferation medium [45]. CECs proliferated and reached confluence while maintaining their characteristics. Human endothelial SFM was broadly used in terms of maintenance media [57,72,73]. Ham's F-12 and M199 supplemented with growth factors were the most common proliferation media [11,31,45,55,72,73]. On the other hand, Opti-MEM-I supplemented with growth factors has also been used in many studies as alternative proliferation basal media [12,30,57].

Human CECs are usually mitotically inactivated and arrested at the G1 phase of the cell cycle. There have been many attempts to overcome the cell cycle arrest using growth factors and other supplements such as insulin, growth factors (FGF), ascorbic acid and serum. This combination aimed to promote CEC proliferation and avoid EndMT.

### L-ascorbic acid 2-phosphate

L-ascorbic acid 2-phosphate (A-2P) is an antioxidation derivative of ascorbic acid and is known to be more stable and effective in stimulating the growth of various cell types than ascorbic acid [74,75]. The study by Shima *et al.* demonstrated the effect of A-2P and FGF-2 on human CEC proliferation *in vitro*. The combination of A-2P and FGF-2 significantly increased the growth and replicative life span of human CECs. The cells were characterized by ICC and real-time (RT)-PCR. Positive staining of human CEC-related markers (ZO-1, CDH2, connexin43 and Na^+^/K^+^-ATPase) was presented using ICC. The mRNAs of voltage-dependent anion-selective channel proteins (VDAC2 and VDAC3), SLC4A4 and chloride channel proteins (CLCN2 and CLCN3) were detected by RT-PCR [76]. The mechanism of A-2P on cell proliferation is not well understood, but upregulation of collagen synthesis and antioxidant activity may be involved. The study in human skin fibroblasts found that supplementation of the medium with A-2P accelerated procollagen processing to collagen and deposition of collagen in the cells, which was confirmed by electron microscopy [75]. This finding was supported by studies in osteoblasts [74], rabbit corneal keratocytes [77] and mesenchymal stem cells (MSCs) [78], which showed that A-2P could promote collagen production. On the other hand, the effect of A-2P on collagen synthesis was investigated using western blot analysis. The result showed that there was no difference in the expression of collagen types I, III and IV between human CECs cultured with and without A-2P. A similar result was obtained by IHC. Further evidence from x-z projection imaging confirmed that A-2P did not promote the collagen accumulation of human CECs [79]. A previous study presented that A-2P protected human CECs against oxidative DNA damage and extended the lifespan of cultured human CECs [76]. A-2P also extended the lifespan of human vascular endothelial cells [80], human skin keratinocytes [81] and cultured human fibroblasts [82]. From these results, the mechanism of A-2P on human CEC proliferation remains unknown. Therefore, further studies are required.

### Basic FGF or FGF-2

FGF-2 is a well-known growth factor used to supplement the culture media of many cell types. Previous studies from Schweigerer *et al.* found the synthesis of FGF-2 in cultured capillary endothelial cells, CECs and lens epithelial cells [83,84]. Moreover, FGF-2 synthesized by these cells stimulated their own proliferation. Thus, it might indicate that FGF-2 acts as an autocrine growth factor in the cells. An *in vitro* study of neuroectodermal and mesodermal origin cells presented that FGF-2 influenced cell growth, migration, differentiation, regeneration and senescence [85,86]. A number of studies demonstrated the ability of FGF-2 to promote bovine, rabbit and human CEC proliferation *in vitro* [21,87,88]. The result from rabbit CEC culture showed that the cells exhibited a polygonal morphology and could reach confluence [88]. A study of human CECs, cultured in FGF-2 containing medium, found a similar result. The cells presented a hexagonal shape and increased proliferation rate [21].

As detailed above, FGF-2 could promote human CEC proliferation. However, the effect of FGF-2 on CECs to EndMT was presented in the study [89]. The cells lost their polarity and presented a fibroblastic phenotype. The EndMT secreted type I instead of type IV collagen and caused retrocorneal membrane formation [89]. Another study showed that IL-1β, secreted by polymorphonuclear leukocytes, induced the production of FGF-2 and transformed CECs to become fibroblastic-like cells through phosphorylation of p27 by PI3-kinase [67]. On the other hand, a recent *ex vivo* corneal endothelium study demonstrated that FGF-2 initiated mesenchymal transition through SNAI1, which induced ZEB1 and CDK2 in parallel [90].

The evidence shows that human CECs cultured in FGF-2 supplemented media express human CEC-related markers. However, there is a chance of CECs being transformed to EndMT and lose their specific markers. Therefore, FGF-2 should be used carefully in the cell culture of CECs.

### ROCK inhibitor

The RAS homologous (Rho) protein family is a member of the RAS superfamily of small guanosine triphosphatases (GTPases). Small GTPases are monomeric proteins and function as a molecular switch in the signal transduction pathway. Rho GTPases are intracellular proteins that regulate the formation of intracellular actin structures in various cell types [91]. Nevertheless, they regulate the function in cellular processes such as cell migration, cell adhesion, cell polarity, wound healing and cytokinesis [92,93]. The specific and essential function of Rho is to promote actin stress fiber formation and focal adhesions [94]. In terms of other biological effects, Rho GTPases are well known to play a primary role in cell cycle progression and in apoptosis. Inactivation of Rho blocks G1–S phase progression in the cell cycle. Meanwhile, an injection of active RhoA into Swiss 3T3 fibroblast quiescent cells induces G1–S phase progression [95]. Although the underlying mechanism has not yet been revealed, the ROCK signaling pathway is suspected of promoting HeLa cell cycle progression [96].

Okumura *et al.* proposed that Y-27632, a selective ROCK inhibitor, promoted cell proliferation and adhesion and inhibited cell apoptosis in CECs [97]. When added to cells, the cells uptake Y-27632 via carrier-mediated facilitated diffusion [98]. Y-27632 bound to the ROCK family of kinase and inhibited their kinase activity by competing with ATP for binding to the catalytic site [99,100]. A study in monkey CECs, cultured in a medium containing 10 μM of Y-27632, demonstrated that Y-27632 promoted cell proliferation and prevented cell apoptosis. The number of Ki-67 positive cells detected by FCM significantly increased at 24 and 48 h post-treatment. Meanwhile, the number of annexin-V positive apoptotic cells was significantly decreased at 24 h [97]. A subsequent study showed that the CECs recovered from the culture plate underwent dissociation-induced activation of Rho/ROCK/MLC signaling, which resulted in cell adhesion impairment. The ROCK inhibitor, Y-27632, enhanced cell adhesion by countering the dissociation-induced activation of this signaling pathway [10].

A preclinical study using a primate corneal endothelial dysfunction model demonstrated regeneration of corneal endothelium following injection of cultured monkey CECs in combination with Y-27632 into the anterior chamber. Transparency was restored to the cornea on day 14 after the injection. In contrast to the injection of monkey CECs without Y-27632, where the cornea remained hazy. The result from IHC showed positive staining of Na^+^/K^+^-ATPase and ZO-1 [10].

Recently, the first-in-man cell-based therapy clinical trial was performed on 11 patients with a diagnosis of bullous keratopathy from FED, pseudophakic bullous keratopathy, postglaucoma surgery and postocular trauma [12]. All of the patients received the same procedure. Briefly, 8 mm from the center of the damaged endothelium was removed using a silicone tip needle without removing the DM. Then, cultured human CECs supplemented with 100 μM of Y-27632 were injected into the anterior chamber. After the procedure, the patients were kept face down for 3 h to ensure that the cells were sunk down on the DM. At 24 weeks after cell injection, the CEC density was no less than 500 cells/mm^2^, and a monolayer sheet of the endothelium was observed. Moreover, the clinical data presented corneal transparency recovery with an improvement of best-corrected visual acuity [12]. After 4 years of follow-up, all 11 patients maintained corneal clarity without any ocular adverse effects, such as graft rejection, graft failure or secondary glaucoma [101].

### CM

CM is harvested from cultured cells. It contains growth factors, metabolites and ECM proteins secreted by the cells into the medium. It can be used to investigate the effect of the mediators produced by that specific cell type on another cell phenotype. For example, growth factors, miRNA and biological molecules can be monitored, and the relationship between these factors and cell phenotype can be identified [102,103].

The effects of CM have been extensively studied. MSC-CM [104], human amniotic epithelial cell (HAEC)-CM [105] and human OASC-CM have been shown to increase human CEC proliferation.

Human CECs cultured in CM obtained from a bone marrow-derived MSC-CM culture were examined and compared with a basal medium. The expressions of fibroblastic markers, collagen types I and IV and fibronectin were determined using RT-PCR. The expression of collagen type IV, which is produced by normal human CECs, was no different between groups. Human CECs cultured in MSC-CM presented significantly higher expression of Ki-67 than that in the basal medium at 15.8 and 10.8%, respectively. Moreover, they expressed functional markers of human CECs, Na^+^/K^+^-ATPase and ZO-1, detected by ICC [104].

The use of HAEC-CM has also been suggested. HAEC-CM helped human CECs maintain the phenotype, enhance proliferative capacity, reduce cell apoptosis, maintain cell-junction formation (ZO-1 and CDH2) and pump human CEC function (Na^+^/K^+^-ATPase) [105].

Another study was performed to determine whether human OASCs had any effect on the proliferation capacity of human CECs *in vitro*. Human CECs were cultured in OASC-CM, and human CEC-related markers were observed. The expressions of human CEC-related markers (CD29, CD105, CD49e, CD166 and vimentin) were detected by FCM; likewise, the expression of Na^+^/K^+^-ATPase, ZO-1, CDH2, Col8a2 and SLC4A4 was detected by ICC and RT-PCR. Human CECs maintained their polygonal cell morphology and proliferative capacity [56].

Several studies demonstrated the addition of various growth factors to promote human CEC proliferation and characterized the cells with human CEC-related markers. If the cells expressed some of these markers, they were assumed to be human CEC-like cells. Human CECs transformed to EndMT were detected by their morphology and loss of polygonal shapes with elongated cell bodies. However, the use of cell morphology may not be the appropriate method for early EndMT detection. At the early stage of fibroblastic transformation, the cells might express some markers produced by fibroblastic cells. EndMT-related proteins and genes might be identified at this stage. The detection of EndMT markers should be performed in human CECs cultured in every media formulated or CM.

## Preservation conditions of CECs

The study of Okumura *et al.* compared the viability of human CECs that were stored using different cryopreservation reagents [106]. Bambanker HRM freezing media were shown to be the most effective, with a cell viability percentage of 89.4%, which is equivalent to 89.2% of cell viability in a group of nonpreserved CECs. The cells proliferated and formed a monolayer cell sheet similar to nonpreserved CECs. Bartakova *et al.* demonstrated the characteristic of CECs after cryopreservation [57]. CECs at passage 0 or 1 were cryopreserved and thawed in different media (human endothelial serum free media [SFM] and Opti-MEM-I). Cell characteristics were evaluated using CD56 as a cell surface marker. The results showed that the expression of CD56 was not different between groups. The cells were further subcultured until passage 10. The expression of CD56 was examined and compared with the cells in passage 1 after thawing and plating for 7 days. The CD56 expression levels were similar between groups [57]. The results from these studies showed the success of cryopreserved human CECs. Preserved cells presented similar cell morphology and characteristics compared with nonpreserved cells and remained stable through several passages.

Other hypothermic storage media were optimized by Wahlig *et al.* [73]. Human CECs were stored in three different storage media (Endo-SFM with serum, Endo-SFM without serum and Optisol-GS) at 23°C and 4°C. Cells were thawed and evaluated for cell viability after 48 h of storage. CECs in Endo-SFM with and without serum showed greater cell viability than that in Optisol-GS in both 23°C and 4°C significantly. After plating and culturing for 2 days, the cell proliferation rate of CECs stored in Endo-SFM with serum at 23°C (1.3% ± 0.6%) was lower than at 4°C (2.6% ± 1.2%). However, there was no statistical significance between groups. Cell characterization was performed in both hypothermic conditions and found that the cells expressed endothelial cell markers, Na^+^/K^+^-ATPase, ZO-1, Prdx-6 and CD166 [73]. From these results, it was assumed that cultured human CECs could be stored in hypothermic conditions while maintaining their characteristics.

## Carriers of endothelial cells for transplantation

Transplanting cultured human CECs is another challenge. Once the cells are cultured, they need a transporting carrier (a sheet or cell suspension). Various cell carriers have been studied and proposed for tissue engineering depending on their properties that should include cytocompatibility, reproducibility, manufacturing possibilities, ease of manipulation in surgery, flexibility, transparency and thickness [107]. Some of which include collagen vitrigels [108], atelocollagen and gelatin hydrogel sheets [109], silk fibroin [63] and tissues such as the xenogeneic substrate of bovine corneal posterior lamellae [110], human anterior lens capsule [111] and amniotic membrane (AM) [53].

AMs have advantages, as they have been widely used in ocular surgery and have already been proven to successfully support the culture of other ocular cells such as limbal epithelial cells [112–115]. However, because of the donor variability between biological materials, degradation rate and sub-optimal transparency, AM has been limited for human CECs. Bioengineering substrates could therefore be advantageous as variability is limited and materials can be selected based on their desirable properties to increase reproducibility and promote mass production when necessary with batch–batch consistency. Although gelatin, collagen hydrogels and silk fibroin mats have been investigated, they were not promising as they lack mechanical strength. Collagen vitrigels have a lengthy process involved in the production [116].

Real Architecture for 3D Tissues (RAFT) biomaterial is composed of type I collagen hydrogel. The methods of production are rapid, simple and reproducible. The hydrogel can be easily manipulated and handled by a surgeon for transplantation [117]. A clear advantage of RAFT is that collagen cross-linking is formed by mechanical compression without any chemical reagent. Thus, cell characteristics and behaviors are not altered.

In addition, analogs of scaffolds to support endothelial cells seem to be promising for developing the optimal cell transplant substrate. Bioactive electrospun scaffolds loaded with drugs to prevent vascularization of the cornea in TE buffer have been investigated [118]. Silk nanofibers loaded with epigallocatechin gallate exhibited a dose-dependent inhibition of human umbilical vein endothelial cell proliferation. Similarly, nanoparticles fabricated by electrospraying to encapsulate biologically active compounds decorated on electrospun membranes are also being investigated. The idea is that the substrate will act twofold to deliver cells to the required area while delivering drugs/growth factors to the eye longterm to maintain transplanted cell health [119].

Despite the advancement in the field of carrier materials, cellular properties and surgical techniques remain to be limiting factors in the success of tissue engineering. Fragile endothelial sheets can now be cultured on a potential scaffold and preloaded in a specific device as a ready-to-use tissue unit. The surgery only requires minimal manipulation and preparation time [120–122]. However, the clinical application of protocols developed *in vitro* is often restricted by the limited proliferative capacity of primary human cell donors. In addition, placing the highly fragile, monolayer endothelial cell sheet into the anterior chamber and firmly fixing it to the posterior cornea pose an unresolved challenge to the surgery. Developing an improved device or a novel surgical technique could alleviate the issue.

## Conclusion & future perspective

A common cause of visual impairment and corneal blindness is corneal endothelial diseases. The standard treatment is corneal transplantation. Unfortunately, the shortage of cornea donors has become a significant problem worldwide. CEC culture is an alternative option to resolve this problem. However, many factors disturb the success of CEC culture, and the variety of donor characteristics is an unavoidable factor. The number of CECs from different donors or different corneas from the same donor is not equal and unpredictable. The age of donors is another factor that has already been declared [40–42]. The number of CECs from young donors is greater than from old donors. The potential of the cell to proliferate is also greater. Nevertheless, CECs from young donors could be passaged while maintaining their characteristics up to passage 4 [42]. However, the corneas from young donors are primarily deployed for transplantation because of high CEC density. The corneas with low CEC density, usually from older donors, are categorized as research-grade corneas [43]. The challenges of culturing CECs from older donors are limited by cell number, low proliferative capacity and easily become senescence [43,123]. Force attachment or accelerating cell attachment methods were proposed as an alternative option for CEC culture from older donors. HA combined with ROCK inhibitor (Y-27632) was used as a force attachment to facilitate CECs from older donors and adhere to the culture plate. CECs with force attachment proliferated and reached confluence faster than the unforced CECs at passage 0 [43]. For passaged cells, the accelerating cell attachment method using viscoelastic solution (Viscoat) was applied in CECs at passage 1. Cell proliferation was enhanced by Viscoat while cell morphology and characteristics were maintained [124]. HA adheres to CEC surface receptors, which are ICAM-1, CD44 and RHAMM, and promotes cell migration [125,126]. A similar property is also present in Viscoat. Proteoglycan at the cell surface can link with Viscoat, which further helps cell adhesion and migration [127]. The benefits of HA and Viscoat could enhance the proliferation rate of CECs from older donors without disturbing cell morphology and characteristics.

Harvesting CECs from the peripheral area of the remained cornea after transplantation can potentially be an optional method [45]. The cells in this area present stem cell markers, which mean that the cells can proliferate and differentiate into mature cells. The number of the cells was dramatically increased regardless if they started from a low cell number [46–49]. In the future, CECs obtained from the PE can become an alternative source of CECs to solve the lacking proliferative capacity issue.

Isolating CECs from DM was a challenging point. The peel-and-digest method is an effective way to obtain CECs. The enzyme type used in the digestion step is of concern. Trypsin [52], dispase and EDTA [53,54] can cause the degeneration of CECs and decrease in cell number. Collagenase seems to be the most effective enzyme [11]. From the authors' experiences, the number of degenerative CECs after exposure to collagenase is lower than other enzymes. Cell plating density is different between studies, and the number ranges from 15,000 to 25,000 cells/cm^2^ [33,57]. This diversity can affect cell characteristic and result reliability. Additionally, CEC morphology changes after plating the cells with a cell number less than 10,000 cells/cm^2^. The cells showed polymegathism and pleomorphism even in passage 0 and could not proliferate. Most CEC studies have been performed in CECs obtained from young donors, and a limited number of studies were from older donors. Thus, CEC plating density from older donors could not be compared. Establishing proper cell plating density will benefit *in vitro* study and further tissue engineering for transplantation.

The characteristic of CECs during culture should be concerned as the supplements in cell culture media, especially FGF-2 can trigger EndMT [67,89]. The cells can lose apical–basal polarity, change in cell shape and acquire mesenchymal characteristics. Several studies proposed that EndMT markers could be used as an early EndMT detection tool before changes in cell morphology occur [30,37]. However, the roles of these proteins on EndMT remain obscure. Future studies on the functions of these cell surface proteins are vital and can guide the way to prevent EndMT.

Considering cell carriers for tissue transplantation, biomaterial and synthetic tissues are developed to reach the goal of biocompatibility. Several types of tissues have been proposed [115,117–119,128]. However, culturing CECs on the material surface and proliferating them while maintaining their specific characteristics are challenging. The combination of tissue engineering and biologically active compound or cell signaling reagents (ROCK inhibitor) could become a new therapeutic option for corneal endothelial diseases [129,130].

Understanding cell biology and characteristics from different conditions of the corneas is essential to improve the proliferation of human CECs. There are several challenges in every step of CEC culture that have already been noted in this review. Thus, recent advances in cell culture and tissue engineering bring hope that effective and safe techniques for treating CEC diseases may soon be developed.

**Executive summary**

- Corneal endothelial cell (CEC) culture is a viable alternative to resolve the global shortage of human donor corneas.
- CECs from old aged donors have limited proliferative capacity and can easily become senescence. However, if induced with growth factors, they can be cultured.
- The use of peripheral CECs of the remaining cornea after transplantation can be an alternative source to collect CECs for cell culture.
- Peel-and-digest method using collagenase is the most successful cell isolation method.
- Cell plating density should be no less than 10,000 cells/cm^2^ to maintain CEC morphology and characteristics from young donors.
- Endothelial-to-mesenchymal transition (EndMT) can be triggered during *in vitro* cell expansion. Cell characterization using CEC markers and EndMT markers should be performed.
- ROCK inhibitor remains the widely used media supplement for the proliferation of human CECs.
- A suitable cell carrier or a scaffold needs to be identified for future transplantation purposes.
